# Caffeine improves biochemical and specific performance after judo training: a double-blind crossover study in a real judo training situation

**DOI:** 10.1186/s12986-021-00544-5

**Published:** 2021-01-23

**Authors:** Kelvin Euton Oliveira Carmo, Diego Ignácio Valenzuela Pérez, Charles Nardelli Valido, Jymmys Lopes dos Santos, Bianca Miarka, Raquel Simões Mendes-Netto, Marina Macedo Rodrigues Leite, Naiara Ribeiro Antoniêtto, Esteban Ariel Aedo-Muñoz, Ciro José Brito

**Affiliations:** 1 Federal University of Sergipe, Aracaju, Brazil; 2 Escuela de Kinesiologia, Magister en Ciencias la Actividad Física y Deportes Aplicadas al Entrenamiento Rehabilitación y Reintegro Deportivo, Universidad Santo Tomás, Santiago, Chile; 3 Laboratory of Psychophysiology and Performance in Sports & Combats, School of Physical Education and Sport, Federal University of Rio de Janeiro, Rio de Janeiro, Brazil; 4 Physical Education Post Graduation Program, Federal University of Juiz de Fora, José Lourenço Kelmer St., Governador Valadares, Martelos, Juiz de Fora, MG 36036-330 Brazil; 5 Sport and Health Sciences Laboratory, Universidad de Santiago de Chile, Santiago, Chile

**Keywords:** Martial arts, Caffeine, Specific task performance, Ergogenic aid, Athletic performance, Fat metabolism

## Abstract

**Background:**

Nutritional ergogenic aids are foods or nutrients that can improve physical performance. Among these foods with ergogenic properties, caffeine has shown that it can increase the fat catabolism, strength, and improve the cognition and time reaction of an athlete, therefore, it is hoped that it can improve the performance of judokas. This study through a double-blind crossover (supplement X placebo) protocol, investigated the effects caffeine supplementation (single capsule containing 5 mg/kg body mass intake 60 min before the session) on biochemical, anthropometrical, physical, subjective and hemodynamic variables measured before, during and after two typical judo trainingcxs sessions (120-min: 40-min of gymnastics; 40-min of specific technics and; 40-min of judo combat).

**Methods:**

8 high-level athletes (21.4 ± 2.0 years; 83.6 ± 15.2 kg; 1.8 ± 0.1 m; 17.9 ± 7.0 Fat%) were evaluated before and after each training for body mass, hydration, upper and lower limb power, performance in the special judo fitness test (SJFT), free fatty acids (FFA) in plasma, uric acid, glucose, lactate, heart rate, and pain. In addition, heart rate, FFA in plasma, uric acid, glucose, lactate, rating of perceived exertion and pain were assessed during the training.

**Results:**

At 120 min, supplementation resulted in a higher concentration of plasma FFA (1.5 ± 0.5 vs. 1.0 ± 0.3 mmol/L; p = 0.047) and lactate (4.9 ± 1.8 vs. 3.0 ± 1.2 mmol/L; p = 0.047), and a lower concentration of uric acid (5.4 ± 0.9 vs. 7.0 ± 1.5 mg/dL; p = 0.04). Supplementation also resulted in performance maintenance (fatigue index) in the SJFT (Δ0.3 ± 2.0 vs Δ1.7 ± 2.5, for caffeine and placebo respectively, p = 0.046). No adverse effects were observed.

**Conclusion:**

Based on the applied dose, intake time, and sample of this study, we can conclude that caffeine produces an ergogenic biochemical effect, and improves performance in judo athletes.

## Introduction

Nutritional ergogenic aids are foods or nutrients that can improve physical performance or accelerate recovery [1]. Among these ergogenic aids, Caffeine ingestion is highly prevalent among combat athletes [2, 3] and this phenomenon has not been studied in judo either of Olympic combats considering real training situations. A full literature review did not demonstrate any research addressing caffeine ingestion and judo performance during the competitive season [4–7]. To obtain information for conditioning and strength training it is important to highlight that open task and intermittent sports training involve complex specific and cognitive skills over a prolonged period of time (120-min), with high-intensity efforts (e.g. attacking with throws, immobilizing, trying to choke or using elbow joint locks) combined with short breaks as well as low-intensity periods. The primary source of energy during a judo match is controversial since previous studies were not able to consider different technical-tactical choices and genetic particularities of athletes during experimental and simulated judo matches [8]. But, investigations agree about the fact that during short and high-intensity efforts, the energy from metabolism comes from creatine phosphate resynthesis and anaerobic glycolysis [9], as a high muscle ATP degradation from glycolysis increases lactate production, affecting anaerobic performance [10]. In this sense, ergogenic aids to preserve body carbohydrate reserves may be a strategy that will improve performance in this modality, since judo requires a high demand for carbohydrates for high performance [2].

Recently, caffeine ingestion has presented a “sparing” effect of carbohydrates, thus improving running performance [11]. Furthermore, in judo, the flexibility of motor control depends on anticipation of subsequent opponent`s action as well as simultaneous and quick decision-making to spatiotemporal modifications, which specific-skills perhaps improve using caffeine, as demonstrated by preceding reports with combat sports [3, 12, 13]. Caffeine supplementation provided a significant difference in activities requiring maximum strength [3]; there was also a lower time-reaction in taekwondo athletes [13]. There is little knowledge about the effect of caffeine on judo performance [2, 14, 15], such studies focused on measuring the isolated effect in the Special Judo Fitness Test [2, 14], demonstrating “unclear” evidence with confounding factors, as varied rapid weight loss processes without dietary control, using the same secondary data or a missing randomized sample and metabolic biomarkers. Bias may complicate efforts to establish a cause-effect relationship between caffeine ingestion during competitive season training to maintain or improve specific skills [2]. Additionally, there is no consensus about the ergogenic effects of caffeine ingestion on muscle strength [16] or combined strength and endurance training [17–19]. There are many adverse effects on sports performance arising from caffeine intake such as tachycardia, increased blood pressure [20], dehydration, diuresis [21], and impaired exercise performance in the heat [22].

It should be noted that judo is a mixed combat sport where the metabolism of elite athletes is divided into 40% aerobic, 8% anaerobic lactic, and 52% anaerobic alactic [23]. Based on these facts, a well-established research design to investigate the ergogenic effects of caffeine ingestion could provide an overview of how this substance affects aerobic and anaerobic systems using specific judo actions and considering volume and intensity of specific training protocols. Once there is an ergogenic effect, nutritionists can establish supplementation strategies within an athlete's diet plan, considering the competition schedule and the need for loss of body mass. Therefore, this study purposed to compare pre and post judo training effects across athletes with caffeine versus placebo intake in specific metabolic biomarkers **(**e.g. uric acid, creatinine, plasma glucose, and lactate serum concentrations) and skill performance (e.g. number of throws, heart rate, recovery heart rate). This investigation hypothesized that judo athletes with caffeine intake present ergogenic effects, increasing FFA, with lower uric acid and lactate concentrations**,** maintaining specific-skills performance.

## Materials and methods

### Participants

Ten highly trained judo athletes (21.6 ± 2.0 years; 83.0 ± 15.1 kg; 1.8 ± 0.1 m; 17.8 ± 6.9 Fat%) were recruited. All participants competed in local, regional, and national competitions (6-regional champion, 3-interstate champion, and 1-national champion) once a month and were regularly training (technical, tactical, and specific physical conditioning training) 4–7 times a week and all they were in the competitive season. Thus, athletes were initially selected according to the following inclusion criteria: (a) training and competing regularly in the past and current year; (b) ≥ 18 years old; (c) sign the Free and Informed Consent Form; (d) completed all phases of this study; (e) ≥ purple belt (≥ 7 years experience in performance training and had experience with official judo events, rules, and procedures used throughout the experimental training). During the two sessions of training, no interventions were made in the food intake or hydration status. Participants were excluded if: (a) they did not complete all stages of the study (n = 1); or (b) there were errors in the data collection (n = 1). Therefore, the final sample was composed of eight judo athletes (21.4 ± 2.0 years; 83.6 ± 15.2 kg; 1.8 ± 0.1 m; 17.9 ± 7.0 Fat%). The procedures performed were previously approved by the Local Research Ethics Committee (Protocol 72368, CAAE: 01723312.2.0000.0058) and followed the Declaration of Helsinki.

### Procedures

**Fig. 1:**
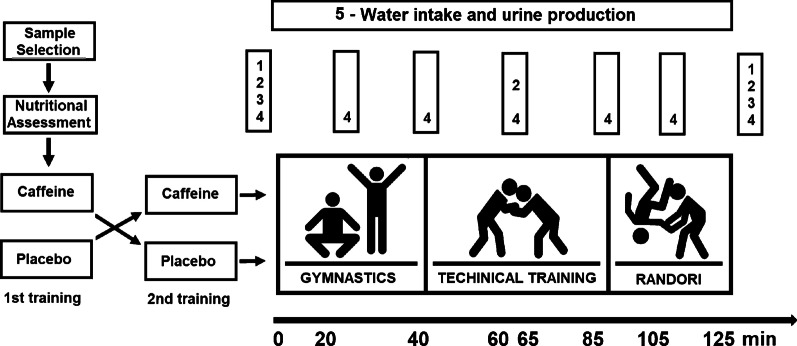
Experimental procedures: 1—anthropometry (body mass, body water, weight, height, blood pressure), 2—blood sample, 3—physical tests (Special Judo Fitness Test, lower and upper limb power), 4—heart rate, rating of perceived exertion and pain

This is experimental applied research, using a controlled, randomized, crossover, double-blind design, separated in three phases: (1) a screening visit (e.g. anamnesis, nutritional assessment, and anthropometric data were collected); (2) a pilot study to determine the sample size, and; (3) the experimental crossover double-blind research with two similar visits, using caffeine (CAF) or a placebo (PLA) intake, the same pre and post-training protocol measures (anthropometry, blood sample, physical tests, heart rate, rating of perceived exertion—RPE, and perceived pain) and identical judo training protocol. Figure 1 summarizes the experimental procedures.

All measures and interventions were realized in air-conditioned judo mat, at a range temperature between 24.5 and 26.5 °C and relative humidity between 63 and 65%. The judo training and measures were performed on the mat, following official training procedures of state and national teams. Furthermore, participants were familiarized with the physical tests, and no capsules were administered. All participants were encouraged to give maximum efforts during the test. They were divided into two groups with a difference of body mass of no more than 10% among selected athletes. First, we performed a representative analysis based on our pilot study (n = 4) and specific literature [2, 14, 15] to determine the appropriate sample size based on the FFA concentration, which was the main indicator for the ergogenic effect of caffeine. It was determined that a minimum sample size of 8 participants would be necessary to achieve 80% statistical power to detect a plasma FFA increase of 1.0 mmol/L throughout the experimental period and 0.5 mmol/L to detect differences among groups (Granmo 5.2; IMIM, Barcelona, Spain). After that, the experimental protocol consisted of four paired analyses of two randomized visits (PRE-PLA × POST-PLA × PRE-CAF x POST-CAF). After the intervention and measurements, these data were compared to verify the impact of judo training, crossing CAF × PLA groups on specific metabolic biomarkers (e.g. uric acid, creatinine, plasma glucose, and lactate serum concentrations) and specific judo skill, and physiologic performance (e.g. number of throws, heart rate, recovery heart rate and with a specific index).

### Diet prescription and supplementation protocol

A dietary assessment was performed two weeks before data collection with a 24-h recall and a food frequency questionnaire being applied, which analyzed the habitual caffeine intake. All were instructed to stop using any supplements and to avoid eating caffeine-containing foods 48-h before the training. The nutritional plan was based and prescribed on self-preferences and aimed to achieve the estimated energy requirement [24] and offer 5–7 g/kg/day of carbohydrates and 1.5–2.0 g/kg/day of proteins [25], however, no foods containing caffeine were prescribed. This procedure aimed to minimize the macronutrient intake differences between subjects, and confounding effects due to the uncontrolled caffeine intake during the experimental period.

All participants were instructed to avoid training 24 h before data collection and rest for at least 8 h. The subjects were asked to eat breakfast at 7:00 a.m. (the breakfast was the same for both conditions). The experiment started at 8:00 am when 10 athletes were randomized to ingest 5 mg/kg body mass of caffeine or placebo in one gelatinous capsule, which is a dosage considered to be safe in previously published papers [17, 26]. The procedures were reversed in the second training, which characterizes the crossover design (Fig. 1). Supplement and placebo capsules were controlled by one of the researchers who did not participate in data collection to ensure the double-blind method of this study. The caffeine and placebo capsules were handled by Belapharma^®^ (batch 0913/13), which is licensed by the Sanitary Federal Government Agency. The training began one hour after the ingestion of the capsules.

### Judo training protocol

Two training sessions were performed separated by four-days (96 h), during the competitive season. The training applied represented a typical training session characterized by a progressive and exhaustive effort according to the protocol described by Brito et al*.* [27]. Each training followed the structure: first, 40 min. of general exercises, followed by 40 min. of technical training and finishing the last stage with 40 min. of combat (*Randori*). Generalized exercise training (gymnastics) consisted of general warm-up exercises, followed by activities involving strength, speed, and aerobic endurance. The technical training was composed of specific movements in judo: throwing techniques (*Nage-waza*), and groundwork (*Katame-waza*). In the Combat stage, the athletes performed bouts without interruptions and points; eight fights were performed (4 min of combat with 1 min of interval). The athletes were permitted to ad libitum water intake throughout the training. The environment temperature was monitored throughout the experimental period. The mean of temperature and relative humidity on the first day were 25 ± 1 ºC and 65 ± 3%, respectively, while on the second day they were 26 ± 1.5 °C and 63 ± 4%, respectively.

### Body composition and hydration state

We performed the anthropometric evaluation according to the standards in Lohman et al*.* [28]. Body mass was measured on a scale with a maximum capacity of 200-kg and a precision of 100-g (Lider P-150 M^®^, São Paulo, BRA). A portable stadiometer measured height with 1-cm accuracy (Auturexata^®^, São Paulo, BRA). Body composition and water volume were estimated by tetrapolar bioelectrical impedance (Biodynamics 310 Model A^®^, Biodynamics Corp., Seattle, USA), with the subject lying in a supine position on a stretcher. All previous orientations recommended for this measurement were followed [29]. Body mass and water volume were measured before and after training. The amount of liquid ingested and the urine produced (collecting bags; Flexor^®^, Rio de Janeiro, BRA) were measured during the training sessions.

### Blood collection and biochemical analysis

Two nurses collected 2 mL of blood from the vein of the antecubital fossa before, in the middle (a pause of 5-min at 60-min of training, Fig. 1), and at the end of the training. From these, 1 mL was collected in a vacuum tube with K_3_ + EDTA anticoagulants (Vacuette^®^, Greiner Bio-one, Campinas, BRA), and the remainder in a coagulant gel tube (Vacuette^®^, Greiner Bio-one, Campinas, BRA). The blood was then centrifuged for 10-min at 3000 rpm to separate plasma (FFA, lactate, and glucose) and serum (creatinine and uric acid). The measurements were performed on the Vitros^®^ 5600 (Ortho-Clinical Diagnostics, Johnson & Johnson Company, Rochester, USA), except for the FFA which were measured in a spectrophotometer (Hitachi U2000^®^, Tokyo, JAP). Biochemical analyses were performed immediately after collection. FFA were used as a marker of lipid catabolism, uric acid as a marker of protein metabolism, and glucose and lactate as carbohydrate metabolism markers.

### Muscular power measures and specific performance of judokas

SJFT—This specific judo test was performed according to indications of Franchini et al*.* [30]. For this, three athletes of similar body mass are needed to perform the SJFT. During this test, the evaluated athlete is called *tori* and the other two partners are called *uke*. Before the test, both *uke* are separated from each other by a distance of six m, and the *tori* is positioned in the middle of them, three m away from each *uke.* This test is composed of three effort moments (15seconds × 30 s × 30 s) with 10 s-break between them. On a signal, the *tori* sprint to one of the partners and employs the throwing technique called *Ippon-seoi-nage* and, consecutively, must complete as many throws as possible within each effort moment, using this technique. The total frequency of completed throws during each period was recorded and the tori’s heart rate (HR) was measured immediately after and 1 min after the test (Polar Team 2^®^, Polar, FIN). Lastly, the SJFT index was calculated by the formula [Index = (HR after + HR 1 min after)/total number of throws].

The reliability of this test factors was reported via the interclass correlation coefficient when evaluating athletes with similar performance levels as those who took part in this study: total number of throws – *0.88*, and index – *0.89*; and a standard error of 2.6% in the total frequency of throws and 4.8% in the index [30].

Countermovement Jump (CMJ)—This test was performed before and after training as a measure of lower limb muscle power using a contact mat (Globus^®^, Rome, ITA) and according to the procedures described by Monteiro et al*.* [31]. Participants were asked to perform the CMJ standing upright with good balance and the trunk as vertical as possible, feet parallel and shoulder-width apart, and, athletes started from an upright standing position and made a preliminary downward movement by flexing the knees and hips, with a knee angle around 90° at the end of the countermovement. The best of three trials was recorded. For the lower limbs, the reliability of this test was reported via interclass correlation coefficient when evaluating athletes with similar performance levels as those who took part in this research, and interclass correlation was *0.96* with a standard error of 2.8% [32].

Pull up power test – The specific upper limb power test measured the upper limb power using an encoder (model PFMA 3010e Muscle Lab System^®^; Ergotest, Langesund, NOR) and according to the procedures described by Fonseca et al*.* [32]. The testing protocol entailed subjects holding a fully flexed shoulder with extended arms for 2 s (to eliminate any slight jumping off the floor, stretch–shortening cycle activity e.g. kipping, or a lack of shoulder flexion) before beginning their pulling action. To ensure a successful repetition, the athletes’ proximal inferior aspect of the mandible must have passed the horizontal plane of the bar. The best of three trials was recorded. The reliability of this test was reported via the interclass correlation coefficient when evaluating athletes with similar performance levels as those who took part in this research, and interclass correlation coefficient was *0.97* with a standard error of 2.5% [32].

### Perceived scales, heart rate, and blood pressure

RPE—the athletes indicated their RPE 6-20 scale [33]. This scale is used to transform athletes’ perceptions of effort into numerical scores between 6 and 20. Athletes were familiarized with the scale in the week before the experimental procedure. Instructions about this scale were read before each experimental session and the participants indicated RPE before, every 20 min, and after training.

Pain Scale—the athletes indicated their rating of perceived pain 0–10 [34]. This scale is used to transform athletes’ perceptions of pain into numerical scores between 0 and 10. Athletes were familiarized with the scale in the week before the experimental procedure. Instructions about this scale were read before each experimental session and the participants indicated their pain before, every 20 min, and after training.

HR was measured with a chest monitor and wristwatch receiver, using the Polar Team II system (Polar^®^, Kempele, SWI). Before the experimental protocol, the participants were fitted with the HR monitor, which was worn before, during, and after judo training and measures. Heart rate measures were obtained before, every 20 min, and after training.

Systolic and diastolic blood pressure were measured by the auscultatory method using a Premium Glicomed^®^ sphygmomanometer (Glicomed^®^, São Paulo, BRA), with the participant sitting with their arm supported at the height of thorax, with systolic and diastolic pressures corresponding to the first and last sounds of Korotkoff, respectively [35].

### Statistical analysis

Normality was verified by the Kolmogorov–Smirnov test and homoscedasticity was observed by the Bartlett criterion, respectively. Descriptive data were presented as mean (SD). The repeated measures ANOVA [condition (PLA × CAF) X time (pre x post-training)] was applied to establish the difference between means. The Mauchly sphericity test and the Greenhouse–Geisser correction (when necessary) were used for validating repeated measurements. The Bonferroni test was adopted post-hoc when a significant difference was observed in ANOVA. The independent sample T-test was used for the water intake and urine production variables. Furthermore, the Eta squared (η^2^) was calculated for the effect size, presenting 0.01 (small), 0.06 (medium) and 0.14 (large). Moreover, Cohen’s d was used to estimate the magnitude of the effect, presenting 0.1 (small), 0.3 (medium), and 0.5 (large) effect size. Lastly, a significance level of p ≤ 0.05 was adopted in all analyzes, and all analyses were performed with the Statistical Package for the Social Sciences (IBM^®^, version 20.0, Chicago, USA).

## Results

**Fig. 2:**
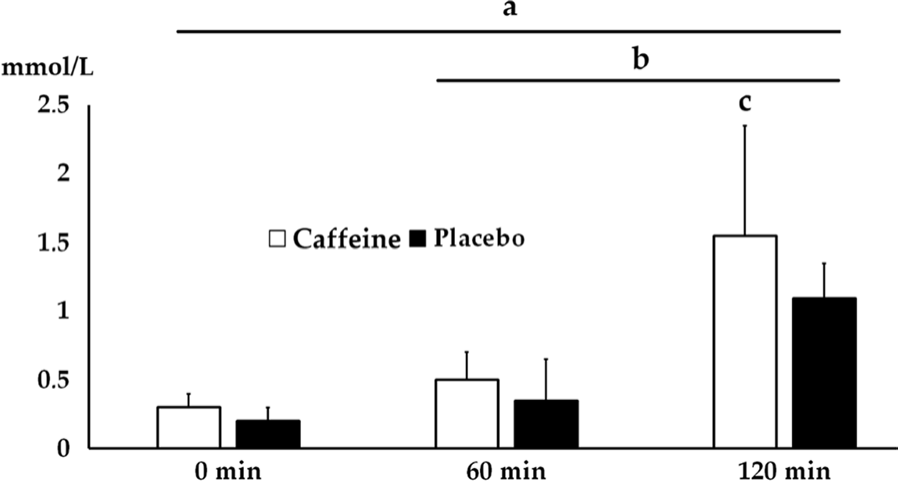
Plasma FFA at the moments before (0 min), during (60 min) and after-training (120 min) for the Caffeine and Placebo Conditions. ^a^*p* = 0.004 0 versus 120 min for both conditions, ^b^*p* ≤ 0.033 60 versus 120 min for both conditions, ^c^*p* = 0.047 Caffeine versus Placebo

Figure 2 shows the plasma FFA concentration. There was a significant interaction between caffeine and moment with a large effect size (F_3,42_ = 4.356; p = 0.038; η2 = 0.46), where the T_120min_ assessment demonstrated higher FFA values than the T_0_ moment (difference = − 1.0 ± 0.4 mmol/L; p = 0.004; 95% CI − 0.2, − 2.1; d = 0.5) and T_60min_ (difference = − 0.8 ± 0.3 mmol/L; p ≤ 0.004; 95% CI − 0.4, − 2.1; d = 0.4). In addition, caffeine supplement showed a higher plasma FFA versus the placebo condition (difference = 0.5 ± 0.6 mmol/L; p = 0.047; 95% CI 0.2,0.10; d = 0.37) after the judo training.

Table 1 presents the results for the serum uric acid and creatinine, as well as plasma glucose and lactate. There was an interaction effect for the serum uric acid with a medium effect size (F_3,42_ = 3.463; *p* = 0.049; η^2^ = 0.23), regardless of the condition, and there was a higher concentration in the measurements obtained at 60 (difference = − 1.1 ± 0.9 mg/dL; *p* = 0.047; 95% CI − 0.3, − 1.4; d = 0.22) and 120 min (difference = − 1.0 ± 1.1 mg/dL; *p* = 0.049; 95% CI − 0.4, − 1.5; d = 0.21). At 120 min there was a significant difference between conditions (difference = − 1.6 ± 1.3 mg/dL; *p* = 0.04; 95% CI − 0.3, − 2.1; d = 0.29), in which the Placebo had a higher serum concentration. We observed a significant effect for serum creatinine (F_3,42_ = 21,897; *p* = 0.003; η^2^ = 0.53) at the measurement moment with a large effect size, where serum concentration was higher during (difference = − 0.3 ± 0.4 mmol/L; *p* = 0.049; 95% CI − 0.1, − 0.6; d = 0.2) and after training (difference = − 0.3 ± 0.3 mmol/L; *p* = 0.049; 95% CI − 0.1, − 0.7; d = 0.2) versus before the training. For plasma glucose, there was no effect of the measurement moment (F_3,42_ = 0.069; *p* = 0.802; η^2^ = 0.12), condition (F_1,14_ = 3.259; *p* = 0.12; η^2^ = 0.1) or interaction (F_3,42_ = 0.682, *p* = 0.54; η^2^ = 0.12). Finally, plasma lactate had an interaction effect with a large effect size (F_3,42_ = 6.439, *p* = 0.04; η^2^ = 0.39), regardless of the condition, and the means measured before were significantly lower than those at 60 (difference = − 12.0 ± 9.3 mg/dL; *p* = 0.045; 95% CI − 8.2, − 22.6; d = 0.29) and 120 min (difference = − 4.2 ± 6.7 mg/dL; *p* = 0.05; 95% CI − 2.2, − 16.6; d = 0.22). In addition, the values measured in the Caffeine Condition were higher in comparison to the Placebo at post-training (difference = − 1.9 ± 2.7 mg/dL; *p* = 0.031; 95% CI − 0.2, − 4.6; d = 0.37).

**Table 1:** Serum concentration of uric acid and creatinine, and plasma glucose and lactate in the Caffeine and Placebo Conditions before, during and post-training

	Before (0 min)	During (60 min)	Post-training (120 min)
*Serum uric acid (mg/dL)*			
Caffeine	4.5 ± 0.9^a^	5.5 ± 1.3	5.4 ± 0.9^c^
Placebo	4.4 ± 0.9^a^	6.3 ± 1.2^b^	7.0 ± 1.5
*Serum creatinine (mg/dL)*			
Caffeine	0.9 ± 0.1^a^	1.2 ± 0.2	1.2 ± 0.2
Placebo	0.9 ± 0.2^a^	1.1 ± 0.3	1.2 ± 0.3
*Plasma glucose (mg/dL)*			
Caffeine	87.1 ± 8.3	99.4 ± 19.8	90.4 ± 19.5
Placebo	84.3 ± 6.4	97.7 ± 24.1	91.9 ± 18.4
*Plasma lactate (mmol/L)*			
Caffeine	1.2 ± 0.9^a^	4.6 ± 2.1	4.9 ± 1.8^c^
Placebo	1.2 ± 0.4^a^	3.4 ± 1.6	3.0 ± 1.2

Data presented by mean ± standard-deviation

^a^*p* ≤ 0.045 from this measurement moment versus the others for the same condition

^b^*p* ≤ 0.049 from this measurement moment versus Post-training for the same condition

^c^*p* ≤ 0.047 versus placebo at the Post-training

Table 2 presents the SJFT results in the Pre and Post-training moments. There was an interaction effect with large and medium effect sizes for the total throws (F_3,30_ = 14.0; *p* ≤ 0.001; η^2^ = 0.32) and fatigue index (F_3,30_ = 5.09; *p* = 0.046; η^2^ = 0.25), where the Placebo presented a lower number of throws (difference = 2.4 ± 1.7; *p* = 0.038; 95% CI 0.5, 5.4.; d = 0.21) and higher fatigue index (difference = − 1.7 ± 2.8; *p* = 0.047; 95% CI − 0.3, − 3.7.; d = 0.2) in the post-training. There was an isolated effect of the measurement moment with a large effect size for the heart rate before (F_3,30_ = 93.286; *p* ≤ 0.001; η^2^ = 0.3) and after training (F_3,30_ = 220.5; *p* = 0.03; η^2^ = 0.24), where the RHR presented a lower mean in comparison to the FHR, regardless of the condition (difference = − 29.1 ± 10.8; *p* = 0.044; 95% CI − 20.3, − 54.8.; d = 0.2, for Before and difference = − 27.4 ± 12.8; *p* = 0.049; 95% CI − 19.9, − 55.2.; d = 0.2, for Post-training).

**Table 2:** Results for the Special Judo Fitness Test (SJFT) before and after-training for the Caffeine and Placebo conditions

Measurement moment	Condition	Total throws	FHR (BPM)	RHR (BPM)	Fatigue Index
Pre-training	Caffeine	22.9 ± 2.8	186.7 ± 6.3	154.1 ± 8.4^a^	15.2 ± 2.3
	Placebo	23.0 ± 2.4	178.1 ± 12.6	151.0 ± 20.8^a^	14.4 ± 1.7
Post-training	Caffeine	22.4 ± 3.7	184.3 ± 7.8	156.3 ± 12.5^a^	15.5 ± 2.0
	Placebo	20.6 ± 2.9^b^	178.3 ± 23.3	152.0 ± 26.9^a^	16.1 ± 2.1^b^

Data presented by mean ± standard-deviation

*FHR* final heart rate, *RHR* recovery heart rate

^a^Significant difference *p* ≤ 0.049 versus FHR

^b^Significant difference *p* ≤ 0.046 versus before for the same condition

There was no significant effect for the lower limbs (Caffeine: 36.6 ± 3.9 and 39.8 ± 5.8 cm; Placebo: 37.7 ± 4.9 and 38.6 ± 4.9 cm; for Before and Post-training moments respectively) of the measurement moment (F_3,30_ = 6.3; *p* = 0.06; η^2^ = 0.12), supplement (F_1,14_ = 0.05; *p* = 0.82; η^2^ = 0.06) or interaction (F_3,30_ = 1.0; *p* = 0.39; η^2^ = 0.09). Similar results (Caffeine: 764.8 ± 93.5 and 793.6 ± 72.2 Watts; Placebo: 768.5 ± 144.6 and 755.8 ± 154.0 Watts; for Before and Post-training moments respectively) were observed for the upper limbs (moment of measurement: F_3,30_ = 0.43; *p* = 0.53; η^2^ = 0.02, supplement: F_1,14_ = 0.34; *p* = 0.72; η^2^ = 0.03, or interaction: F_3,30_ = 0.23; *p* = 0.8; η^2^ = 0.01).

**Fig. 3:**
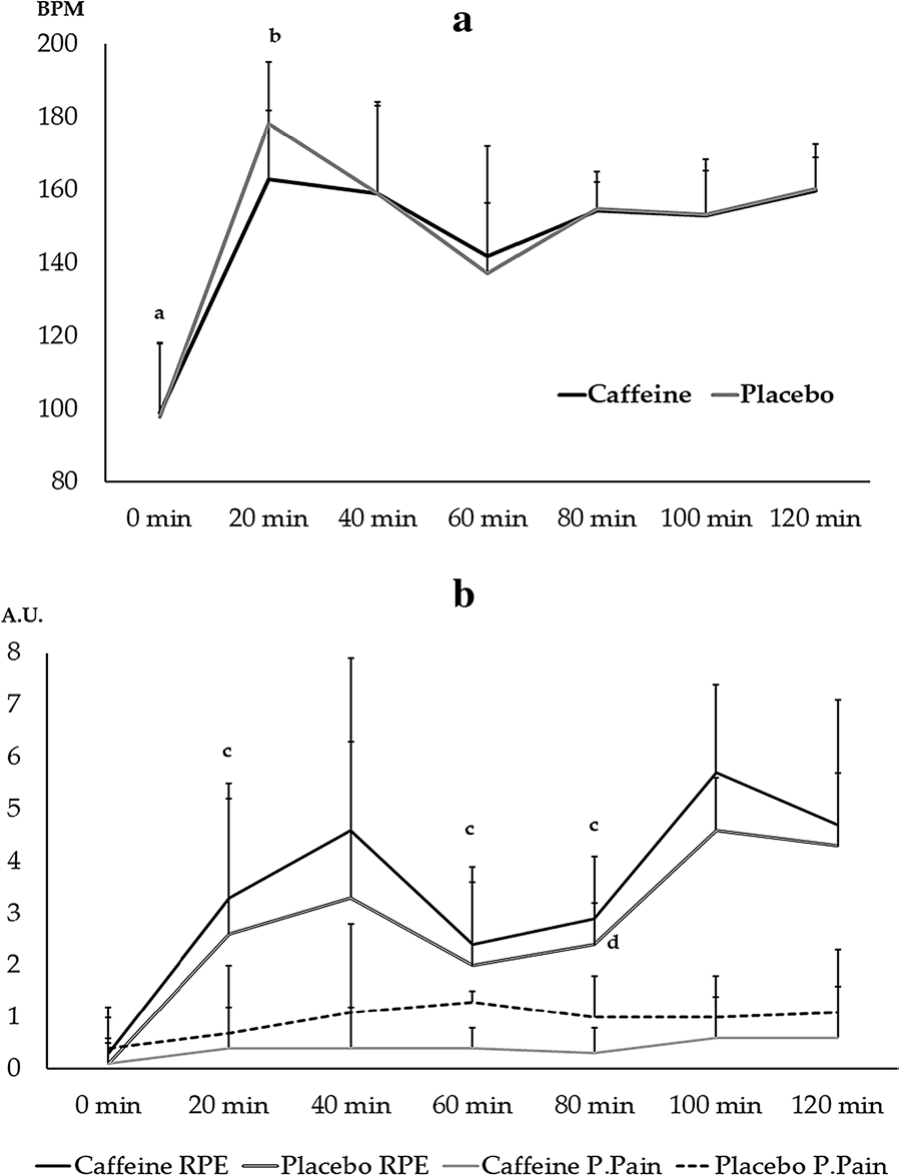
Heart rate (**a**), rating of perceived of exertion and pain (**b**) in the Caffeine and Placebo conditions throughout the training. RPE—ratting of perceived exertion; P.Pain—perceived pain. ^a^*p* ≤ 0.48 for this measurement moment s. 20, 40, 80, 100 and 120 min (regardless condition). ^b^*p* = 0.041 for this measurement moment versus 60 min for the Placebo condition. ^c^*p* ≤ 0.05 for this measurement moment versus 100 min for Caffeine. ^d^*p* ≤ 0.044 for this measurement moment versus 80 min and 100 min for Placebo

**Fig. 4:**
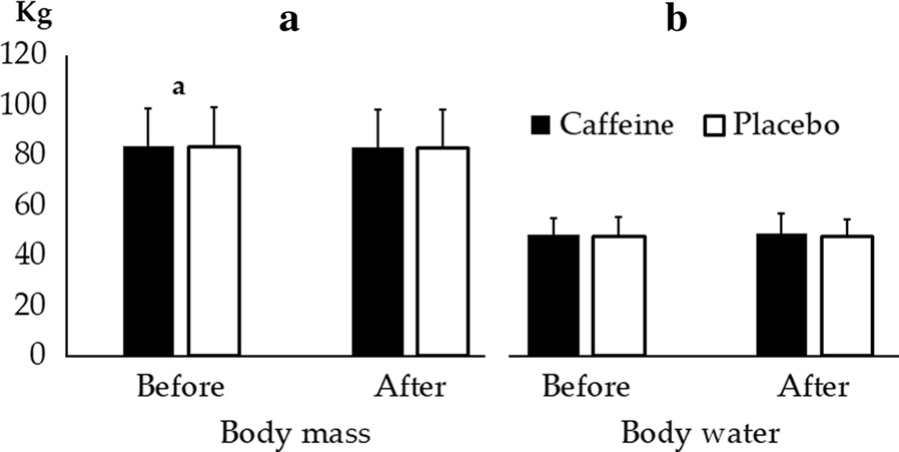
Body mass (**a**) and Body water (**b**) before and after training for Caffeine and Placebo conditions. a *p* ≤ 0.001 versus after moment regardless condition

There was no significant effect for systolic blood pressure (125.7 ± 7.9 and 130.0 ± 10.0 for Caffeine and Placebo at Before, respectively, 127.1 ± 11.1 and 122.9 ± 15.0 for Caffeine and Placebo at Post-training, respectively) of the measurement moment (F_3,30_ = 1.33; *p* = 0.27; η^2^ = 0.1), supplement (F_1,14_ = 1.98; *p* = 0.24; η^2^ = 0.09) or interaction (F_3,30_ = 3.0; *p* = 0.11; η^2^ = 0.2). Similar results were observed for the diastolic blood pressure (80.0 ± 8.2 and 82.9 ± 12.5 for Caffeine and Placebo at Before, respectively, 81.4 ± 13.5 and 82.9 ± 11.1 for Caffeine and Placebo at Post-training, respectively), as there was no significant effect of the measurement moment (F_3,30_ = 0.58; *p* = 0.81; η^2^ = 0.01), supplement (F_1,14_ = 0.68; *p* = 0.7; η^2^ = 0.01) or interaction (F_3,30_ = 0.6; *p* = 0.8; η^2^ = 0.01). Figure 3 showed the results for the heart rate, rating of perceived exertion, and perceived pain. We observed an isolated effect for the heart rate of the measurement moment with a large effect size (F = 34.817; *p* ≤ 0.001; η^2^ = 0.68) (Fig. 4).

There was a significant effect for rating of perceived exertion (F = 8.49; *p* = 0.04; η^2^ = 0.58) of the measurement moment (the differences are shown in Fig. 3). However, there was no measurement moment effect (F_3,105_ = 2.23; *p* = 0.18; η^2^ = 0.13), supplement (F_3,105_ = 0.71; *p* = 0.53; η^2^ = 0.19) or interaction (F_3,105_ = 0.5; *p* = 0.65; η^2^ = 0.014) for perceived pain. The differences are shown in Fig. 3.

There was isolated effect for the body mass of the measurement moment with a large effect size (F_3,30_ = 29.68; *p* ≤ 0.001; η^2^ = 0.71), where the post-training means was lower than the Before moment (difference = − 0.6 ± 0.2; *p* ≤ 0.001; 95% CI − 0.9, − 0.4). There was no effect for body water (48.2 ± 6.5 and 47.5 ± 7.7 for Caffeine and Placebo at Before, respectively, 49.0 ± 7.8 and 47.6 ± 6.7 for Caffeine and Placebo at Post-training, respectively) of the measurement moment (F_3,30_ = 0.78; *p* = 0.39; η^2^ = 0.06), supplement (F_1,14_ = 0.15; *p* = 0.8; η^2^ = 0.05) or interaction (F_3,30_ = 0.46; *p* = 0.46; η^2^ = 0.04).

There were no significant differences for water intake between conditions (2.9 ± 0.9 and 2.7 ± 0.2 L for Caffeine and Placebo, respectively T_1,15_ = 0.41; *p* = 0.69; d′ = 0.02). Similar results were observed for urine production (0.3 ± 0.1 and 0.2 ± 0.1 L for Caffeine and Placebo, respectively T_1,15_ = 1.47; *p* = 0.17; d′ = 0.1).

## Discussion

Caffeine supplementation has shown an ergogenic effect in many sports competitions [1], however, the results in intermittent sports are inconsistent, and in some cases even result in ergogenic effect [19]. To the authors’ knowledge, this is the first study that has measured caffeine supplementation before judo training, verifying ergogenic and performance effects. The main results indicated that the supplemented group showed a higher plasma FFA concentration and a lower concentration of uric acid in the post-training. Compared to the Before moment, the Placebo presents lower total throws and higher fatigue index on the SJFT at the Post-training moment.

Plasma FFA concentrations showed a moderate increase after 60 min of judo training, with a mean rise of 30% above the initial concentration (Fig. 1). The response was significantly higher after 120 min of judo training when it was observed an interaction with the caffeine ingestion, demonstrating ~ 50% more FFA concentration than in placebo condition. This difference can be explained by the fact that xanthines of caffeine manifest a pronounced effect on adipose tissue metabolism [36] in interaction with judo training. Preceding reports indicated that cyclic 3′,5′ adenosine monophosphate promotes lipolysis in adipose tissue. The enzyme, phosphodiesterase, which antagonizes the activity of cyclic 3′,5′ adenosine monophosphate is inhibited by caffeine and other xanthines. As a consequence, the concentration of 3′5′ adenosine monophosphate in adipose tissue rises with an enhanced lipolysis effect. Studies so far indicate that catecholamines also increase the concentration of cyclic 3′5′adenosine monophosphate in adipose tissue; however, per a different mechanism, mediating the cyclic 3′5′adenosine monophosphate increases on cellular lipase and lipolysis.

An unexpected result is the increase of FFA difference at 60 min and 120 min moments, comparing placebo and caffeine conditions. Endurance training results in decreased plasma FFA turnover and oxidation during a 90 to 120 min session of submaximal continuous exercise because of a slower rate of FFA from adipose tissue [37]. This difference between continuous and intermittent intervention highlight present results, caffeine could promote an increase ergogenic aid effect in interaction with intermittent training, as judo. It is in agreement with Lopes-Silva et al*.* [15], who showed that intermittent activities with caffeine intake generate more FFA concentration, decreasing perceived pain through blocking central adenosine receptor. Therefore, the persistence of FFA mobilization after blocking agents and adrenalectomy in judo athletes supports this dual result. Moreover, caffeine increases the availability of FFA to be catabolized, resulting in spare glycogen [11]. In our results, the supplemented judoka showed a higher plasma FFA concentration after-training, while there was a concomitant increase in serum uric acid production in the placebo condition. Serum uric acid is a product of purine metabolism, as increased blood concentrations have been observed as a result of increased protein catabolism [38]. Strenuous aerobic exercise results in increased uric acid production [39], and possibly the supplemented group demanded lower protein catabolism because they used higher amounts of fatty acids. It is also important to note that the supplemented group had a higher lactate concentration after training, which is an indicator of carbohydrate catabolism [40] since this group possibly catabolized more lipids and spare carbohydrates during the training, allowing the supplemented condition to catabolize a higher amount of carbohydrates in the high-intense moments of the training (*randori*) and SJFT.

Preceding reports [41, 42] indicated a significant correlation between HR and RPE during a judo competition (r = 0.88, *p* = *0.05*) and in a sequential simulated training with four combats (*randori*) when observed the second (r = 0.70, *p* = *0.05*) and the third *randori* (r = 0.64, *p* = *0.05*) [7]. Present results indicated similar results during *randori* (80–120 min) maintaining an intermittent exercise ~ 82% of HR_max_, classified as intense. Findings indicated that HR-RPE correlation differentiates with caffeine condition in initial moments – HR-T_20min_ when caffeine had ~ 10% beats per minute less than placebo condition, but with similar RPE. Caffeine could influence HR during moments of efforts (i.e. judo training and SJFT) and in rest moment (i.e. SJFT) with doses between 6 and 9 mg/kg body mass, while 4 mg/kg body mass and 6 mg/kg body mass after rapid weight loss in judo did not increase significantly HR, as present results [2, 15]. Using Edwards’ internal training load (eTRIMP) monitoring conversion [43] by measuring a product of the accumulated training duration (minute) of 5 zones by a coefficient related to each zone it is possible to suggest a dose–response relationship between training load and changes in performance associated with caffeine intake: In the present result, after the warm-up, athletes with caffeine maintained their internal load between the 3rd and the 4th zones eTRIMP (HR range 72% to 82%HR_máx_, with ∆ of 10%), while in placebo situation with similar training condition they had an expanded eTRIMP, using between the 3rd and 5th zones (HR_mean_ range 69% to 90%HR_máx_, with ∆ of 21%) [7]. This result could be used in practical applications and future studies to understand the mechanisms involved in self-regulation during a judo championship, using caffeine and placebo conditions.

Moreover, the findings highlight that caffeine ergogenic effect was able to maintain SJFT performance and fatigue index after 120 min of judo training in the 3rd and 4th zones of training, while Placebo decreases the number of throws, increasing the fatigue index. Similar to our results, other studies have also shown an ergogenic effect of caffeine on the SJFT [2, 14]. In contrast, caffeine did not result in an ergogenic effect on muscle power. Our results do not corroborate the findings of Del Coso et al*.* [5], who observed an increase in lower (back-squat) and upper limb power (bench-press). However, it is emphasized that this protocol measured power alone. In addition, our results did not indicate a hypoalgesia effect, being in contrast to the findings of Green et al*.* [22] who observed higher strength performance until failure, possibly associated with lower perceived pain and effort. Caffeine is hypothesized to increase the coupling of the actin and myosin bridges, resulting in lower perceived pain and high strength [20].

Present caffeine doses of 5 mg/kg body mass are related with optimal doses, reducing RPE and perceived pain, this substance rises the activation of the sympathetic central nervous system over adenosine receptor antagonism—blocking the inhibitory properties of endogenous adenosine at A_1_ and A_2A_ receptors and subsequently the perceived pain [44]. Consequently, glutamate, norepinephrine, and dopamine release, reducing RPE [44]. Recent research with female karate athletes indicated that reduction of RPE and pain perception in physical fitness tests using 5 mg/kg body mass, while the dose of 2 mg/kg body mass was not able to confer any supplementary enhancement in performance. In agreement, preceding reports with judo athletes demonstrated caffeine effects in the SJFT performance—authors showed that doses between 4 and 9 mg/kg are able to improve the total frequency of throws [2, 15]. However, other studies did not show any effect using 6 mg/kg [14]. These differences could arise from different methodological approaches, as Felippe et al*.* [14] had an alternative pre-supplementation during the recovery moment from a 5-day weight-loss period and observed a combination between placebo, caffeine and sodium bicarbonate with − 120 min, − 90 min and − 60 min before the SJFT.

Regarding adverse effects, some studies with caffeine supplementation also have ergolytic [45] or null effects [19]. Adverse effects are generally observed at doses above 9 mg/kg BM [26, 45]. Studies have shown that caffeine consumption results in tachycardia, hypertension [20], dehydration [21], and increased rating of perceived exertion [22]. However, it is emphasized that the dosage used in the present study is considered safe [1, 13, 26, 45]. In fact, there was no difference between the conditions for heart rate, systolic and diastolic blood pressure, rating of perceived exertion, water, and body mass. In agreement with these results, Armstrong et al*.* [21] state that caffeine does not result in excessive dehydration, hyperthermia, or impaired performance for exercise in hot conditions. Our results also corroborate the findings of Del Coso et al*.* [4], who observed that caffeine does not increase dehydration or diuresis, even when exercise is performed in hot environments, as the release of the antidiuretic hormone during exercise is possibly more potent than the diuretic action of caffeine [46]. Nevertheless, we emphasize that there is no scientific data to support the safety and effectiveness of chronic caffeine supplementation [26].

The present study showed that caffeine can be beneficial without observable adverse effects. Thus, our results can be applied by nutritionists to improve the performance of athletes, especially when the aim is long-term weight loss and improved performance at specific periods of preparation for competition. We applied a controlled test protocol during judo training. However, it should be noted that our study is limited to male athletes, and differences in the effects of combined exercise supplementation may be different between men and women [47]. We also did not use spirometry to estimate the metabolic contribution of macronutrients and energy systems as previously applied in other studies in this combat sport [48, 49], but it is not possible to perform this measurement in the experimental manner of our study. We also recognize that even though our study showed a sample size calculation, this may be a limitation of our protocol. Our results represent an advance over previous studies that supplemented caffeine in judokas, and we believe that future studies should investigate the use of caffeine to promote weight loss and its use associated with competitive performance. In addition, other biochemical variables can be measured such as anti-oxidants enzymes, immune cells, and hormones.

## Conclusions

Based on the established objectives, applied methods, and results obtained, we conclude that the supplementation of 5 mg/kg BM resulted in higher plasma fatty acids and lactate, lower concentration of serum uric acid, lower fatigue index, and a high number of throws on the SJFT. As possible practical applications of the present study, caffeine consumption may increase the performance of judokas in training, increasing athletic ability during the final part of the training in which the combat sessions (*randori*) are performed. Furthermore, good performance in *randori* is important because it is the type of training which is most similar to the competition.

## Data Availability

The data used to carry out the present study are allocated in spreadsheets and, if necessary, they will be made available for public consultation.

## Abbreviations

SJFT: Special judo fitness test
FFA: Free fatty acids
ATP: Adenosine triphosphate
CAF: Caffeine
PLA: Placebo
PRE: Pre-training
POST: Post-training
BRA: Brazil
USA: United States of America
JAP: Japan
FIN: Finland
EDTA: Ethylenediamine tetraacetic acid
HR: Heart rate
CMJ: Countermovement jump
NOR: Norway
RPE: Rating of perceived exertion
ANOVA: Variance analysis
FHR: Final heart rate
RHR: Recovery heart rate
eTRIMP: Edwards’ internal training load
